# Bicuspid aortic valve morphology and aortic valvular outflow jets: an experimental analysis using an MRI-compatible pulsatile flow circulation system

**DOI:** 10.1038/s41598-021-81845-w

**Published:** 2021-01-22

**Authors:** Kaoru Hattori, Natsuki Nakama, Jumpei Takada, Gohki Nishimura, Ryo Moriwaki, Eita Kawasaki, Michinobu Nagao, Yasuhiro Goto, Hiroshi Niinami, Kiyotaka Iwasaki

**Affiliations:** 1 Cooperative Major in Advanced Biomedical Sciences, Joint Graduate School of Tokyo Women’s Medical University and Waseda University, Waseda University, Tokyo, 1628480 Japan; 2 Department of Cardiovascular Surgery, Tokyo Women’s Medical University, Tokyo, 1628666 Japan; 3 Department of Integrative Bioscience and Biomedical Engineering, Graduate School of Advanced Science and Engineering, Waseda University, Tokyo, 1628480 Japan; 4 Department of Modern Mechanical Engineering, Graduate School of Creative Science and Engineering, Waseda University, Tokyo, 1628480 Japan; 5 Department of Diagnostic Imaging and Nuclear Medicine, Tokyo Women’s Medical University, Tokyo, 1628666 Japan; 6 Department of Radiological Service, Tokyo Women’s Medical University Hospital, Tokyo, 1628666 Japan

**Keywords:** Cardiology, Diseases, Engineering

## Abstract

The characteristics of aortic valvular outflow jet affect aortopathy in the bicuspid aortic valve (BAV). This study aimed to elucidate the effects of BAV morphology on the aortic valvular outflow jets. Morphotype-specific valve-devising apparatuses were developed to create aortic valve models. A magnetic resonance imaging-compatible pulsatile flow circulation system was developed to quantify the outflow jet. The eccentricity and circulation values of the peak systolic jet were compared among tricuspid aortic valve (TAV), three asymmetric BAVs, and two symmetric BAVs. The results showed mean aortic flow and leakage did not differ among the five BAVs (six samples, each). Asymmetric BAVs demonstrated the eccentric outflow jets directed to the aortic wall facing the smaller leaflets. In the asymmetric BAV with the smaller leaflet facing the right-anterior, left-posterior, and left-anterior quadrants of the aorta, the outflow jets exclusively impinged on the outer curvature of the ascending aorta, proximal arch, and the supra-valvular aortic wall, respectively. Symmetric BAVs demonstrated mildly eccentric outflow jets that did not impinge on the aortic wall. The circulation values at peak systole increased in asymmetric BAVs. The bicuspid symmetry and the position of smaller leaflet were determinant factors of the characteristics of aortic valvular outflow jet.

## Introduction

Bicuspid aortic valve (BAV) is the most prevalent congenital heart malformation; it occurs in 0.5–2% of the population^1^. Up to 80% of patients with BAV develop ascending aortic dilatation, called aortopathy, with serious clinical sequelae, such as 6–9 times increased risk of aortic dissection^1–4^. The natural history of aortopathy in BAV is quite different from that in tricuspid aortic valve (TAV). Progression of aneurysmal diameter is significantly faster with BAV than with TAV, even after aortic valve replacement^5,6^, possibly due to underlying intrinsic medial fragility combined with long-term exposure to BAV-related hemodynamic alterations. Therefore, the current guidelines on aortic diseases generally recommend early surgical intervention in a significantly dilated ascending aorta with BAV—defined as maximum diameter > 45 mm—to prevent sudden death from aortic dissection or rupture^7–9^. However, the treatment of a mildly dilated ascending aorta (40–45 mm) at the time of aortic valve surgery remains controversial due to the markedly heterogeneous characteristics of aortopathy in BAV^10,11^.

With the recent improvements in pre-procedural imaging and devices, transcatheter aortic valve implantation (TAVI) is considered a safe and feasible treatment option for aortic valve stenosis with BAV^12^. Furthermore, it has been reported that TAVI could be performed safely in patients with a mildly dilated ascending aorta and low intraprocedural risk^13^. Given the expanding indications for TAVI in patients with BAV, individualized strategies to manage BAV based on a reliable index for the prediction of the risk of aortopathy are required.

It is being increasingly recognized that aortopathy in BAV is affected by valve-related hemodynamic alterations. Recent studies based on time-resolved three-dimensional (3D) phase contrast magnetic resonance imaging (4D-flow MRI) have demonstrated that BAV morphology affects the eccentricity of the outflow jet and helical systolic flow, which are related to aortic wall remodeling^14,15^. However, due to several cofactors that affect the aortic hemodynamics, such as age, blood pressure, valvular function, and dimensions of the aorta, the relationships between BAV morphology and hemodynamics are not well understood clinically. This study aimed to elucidate the effects of BAV morphology on the characteristics of aortic valvular outflow jet using an MRI-compatible pulsatile flow circulation system to identify BAV phenotypes at high risk of aortopathy.

## Methods

### Tissue-based aortic valve models

Three types of aortic valve models with annulus diameter of 24 mm, including TAV, asymmetric BAV (ABAV), and symmetric BAV models, were prepared using bovine pericardium and a short segment of bovine thoracic descending aorta. The tissues were obtained from a local abattoir. The followings were three steps in devising the aortic valve model: (1) preparation of the bovine pericardium and a short segment of the aorta; (2) creating pericardial valve leaflets; and (3) suturing the pericardial leaflets to a tubular aorta. Preparation of the bovine pericardium and aorta began with manual removal of fat on their outer surfaces. The pericardium was rinsed using physiological saline solution and not treated with glutaraldehyde. The bovine aorta was excised to prepare a short segment of the tubular aorta with an inner diameter of 24 mm and length of 10 cm. The intercostal arteries branching from it were ligated with 5–0 monofilament.

**Figure 1:**
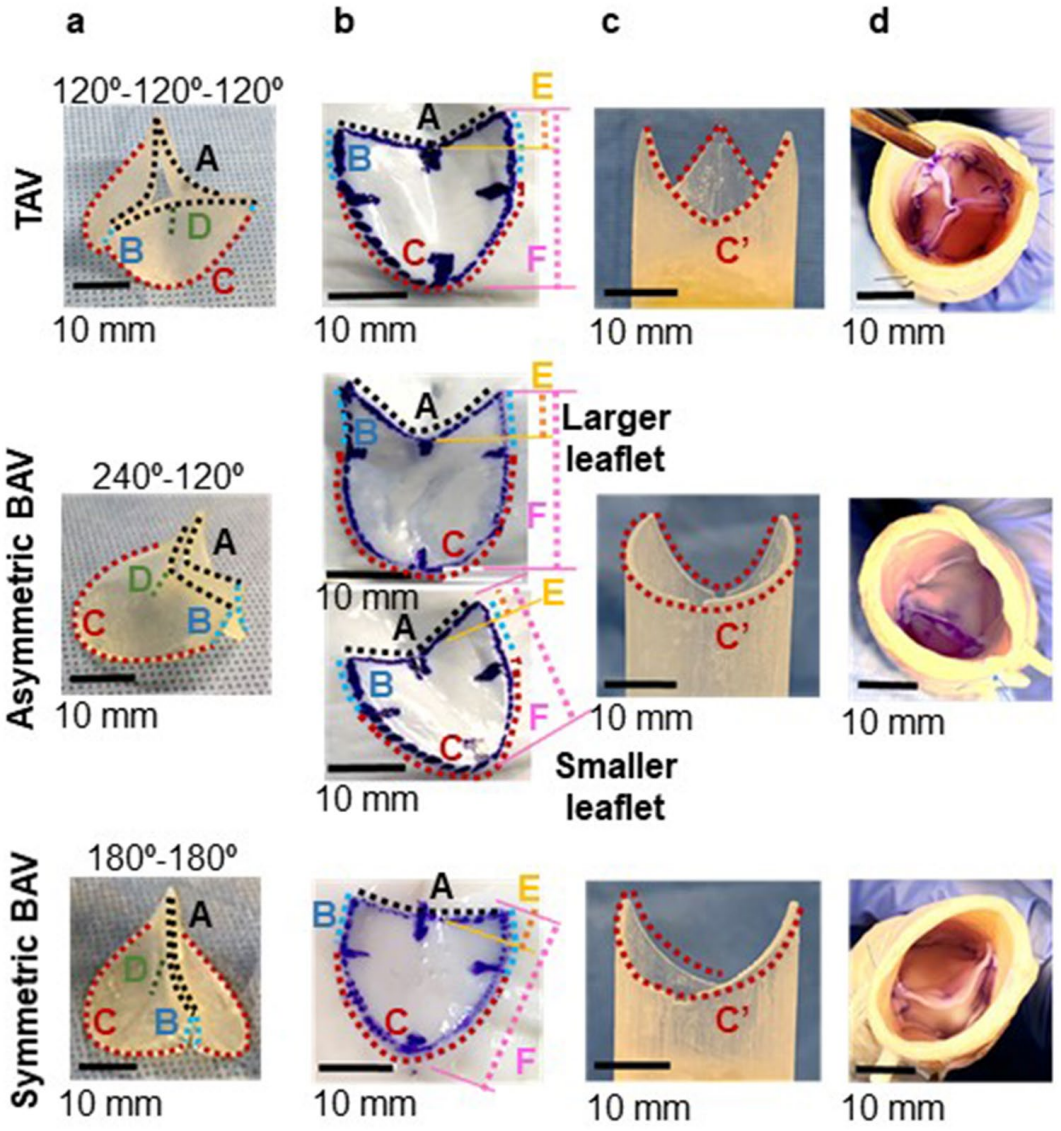
The devices and procedures used in preparing the aortic valve models. (**a**) Morphotype-specific leaflet-trimming three-dimensional templates for preparing pericardial leaflets. (**b**) Pericardial leaflets in tricuspid aortic valve (TAV), asymmetric bicuspid aortic valve (ABAV), and symmetric BAV models. Bovine pericardium is pressed on the templates and cut along their edges. (**c**) Morphotype-specific leaflet-suturing guides. Annular margins of pericardial leaflets (C) are sutured to the aortic wall along the annular curve of the suturing guide (C′). (**d**) TAV, ABAV, and symmetric BAV models with annular diameters of 24 mm. A: free margin; B: commissural margin (length of 5.2 mm); C: annular margin; C′: annular curve; D: coaptation height (length of 6.2 mm); E: depth of the central portion of the leaflets from the upper edge of the commissures (length of 2.7 mm); F: leaflet length (length of 20 mm).

Morphotype-specific valve-devising apparatuses, complementary pair of a leaflet-trimming template, and leaflet-suturing guide were developed to prepare the aortic valve models. Valve-devising apparatuses were designed using computer-aided design (CAD) software (Solidworks 2019, Dassault Systemes Solidworks Corporation, Paris, France) using the following data regarding the geometry of the aortic cusp: annulus diameter, 24 mm; coaptation height, 6.2 mm; commissural length, 5.2 mm; leaflet length, 20 mm; and depth of the central portion of the leaflets from the commissures, 2.7 mm (Fig. 1). The coaptation height of the aortic valve was based on a previous study in healthy humans^16^. The values of the commissural length and the depth of the central portion of the leaflets from the commissures were determined based on the average values in the porcine aortic root. Seven porcine aortic roots with an annulus diameter of approximately 24 mm (mean annulus diameter: 24 ± 0.4 mm, n = 7) were obtained from the local abattoir. Using an X-ray micro-computed tomography (CT) (TDM1300-IS, Yamato Scientific, Tokyo, Japan), the geometric valves were measured under the condition that the aortic valves were closed under the static pressure of 30 mmHg. The cusp angles were defined as 120°–120°–120°, 240°–120°, and 180°–180° in TAV, asymmetric BAV, and symmetric BAV models, respectively (Fig. 1a). CAD data were exported to a 3D printer (Eden260vs, Stratasys, Valencia, CA, USA) and modeled using acrylic resin (FullCure720, Altech, Tokyo, Japan).

In the valve-devising procedure, the leaflets were first cut from the bovine pericardium using a morphotype-specific leaflet-trimming 3D template (Fig. 1a). The bovine pericardium was pressed on the template and cut along the edge with 1-mm seam allowance (Fig. 1b). Next, a complementary morphotype-specific leaflet-suturing guide (Fig. 1c) was inserted into the short segment of the tubular aorta. The annular margins of the leaflets were sutured with 5–0 monofilament continuous sutures to the aortic wall along the annular curve of the leaflet-suturing guide. The commissural margins of adjacent leaflets were sutured together with interrupted sutures and attached to the aortic wall (Fig. 1d).

### Classification scheme of BAV morphology

**Figure 2:**
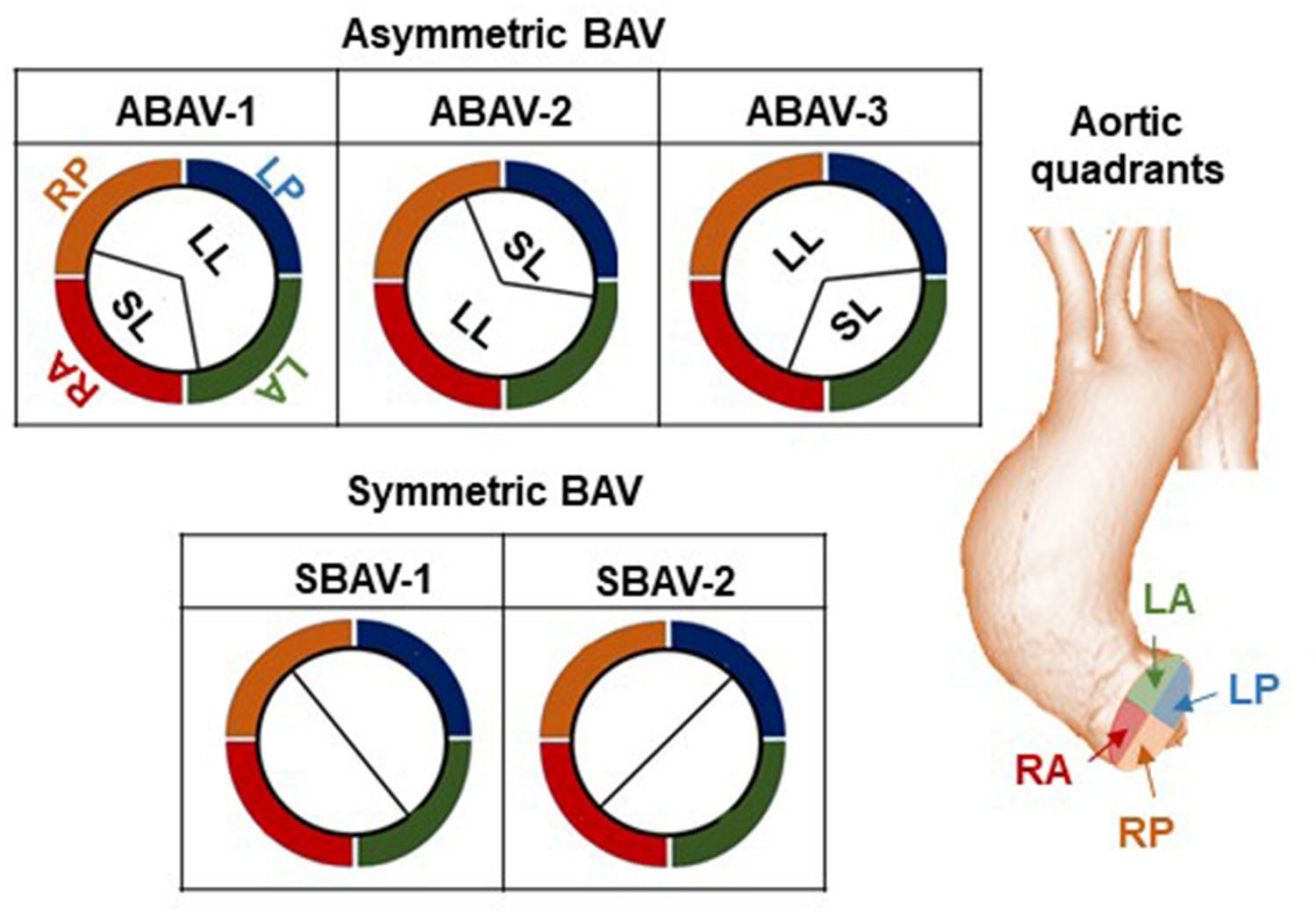
A classification scheme for bicuspid aortic valve (BAV) morphology based on the symmetry and position of the leaflets. Asymmetric BAVs (ABAVs), with two cusp angles of 240°–120°, includes ABAV-1, ABAV-2, and ABAV-3 morphologies. Symmetric BAVs, with two cusp angles of 180°–180°, includes SBAV-1 and SBAV-2 morphologies. The spatial positions of the leaflets are defined based on the aortic quadrant that the leaflets face. *LA* left-anterior quadrant of the ascending aorta, *LP* left-posterior quadrant of the ascending aorta, *RA* right-anterior quadrant of the ascending aorta, *RP* right-posterior quadrant of the ascending aorta, *LL* larger leaflet, *SL* smaller leaflet.

The classification of BAV morphology was based on two characteristics: the symmetry and position of the leaflets (Fig. 2). The spatial position of the leaflets was defined based on the aortic quadrant faced by the leaflets. ABAV models included three morphologies (ABAV-1, ABAV-2, and ABAV-3); they included the smaller leaflet facing the right anterior (RA), left posterior (LP), and left anterior (LA) quadrants of the ascending aorta, respectively. Symmetric BAV models included two morphologies (SBAV-1 and SBAV-2) with symmetric leaflets facing RA-LP and LA-right posterior (RP) quadrants of the ascending aorta, respectively.

### MRI-compatible pulsatile flow circulation system

**Figure 3:**
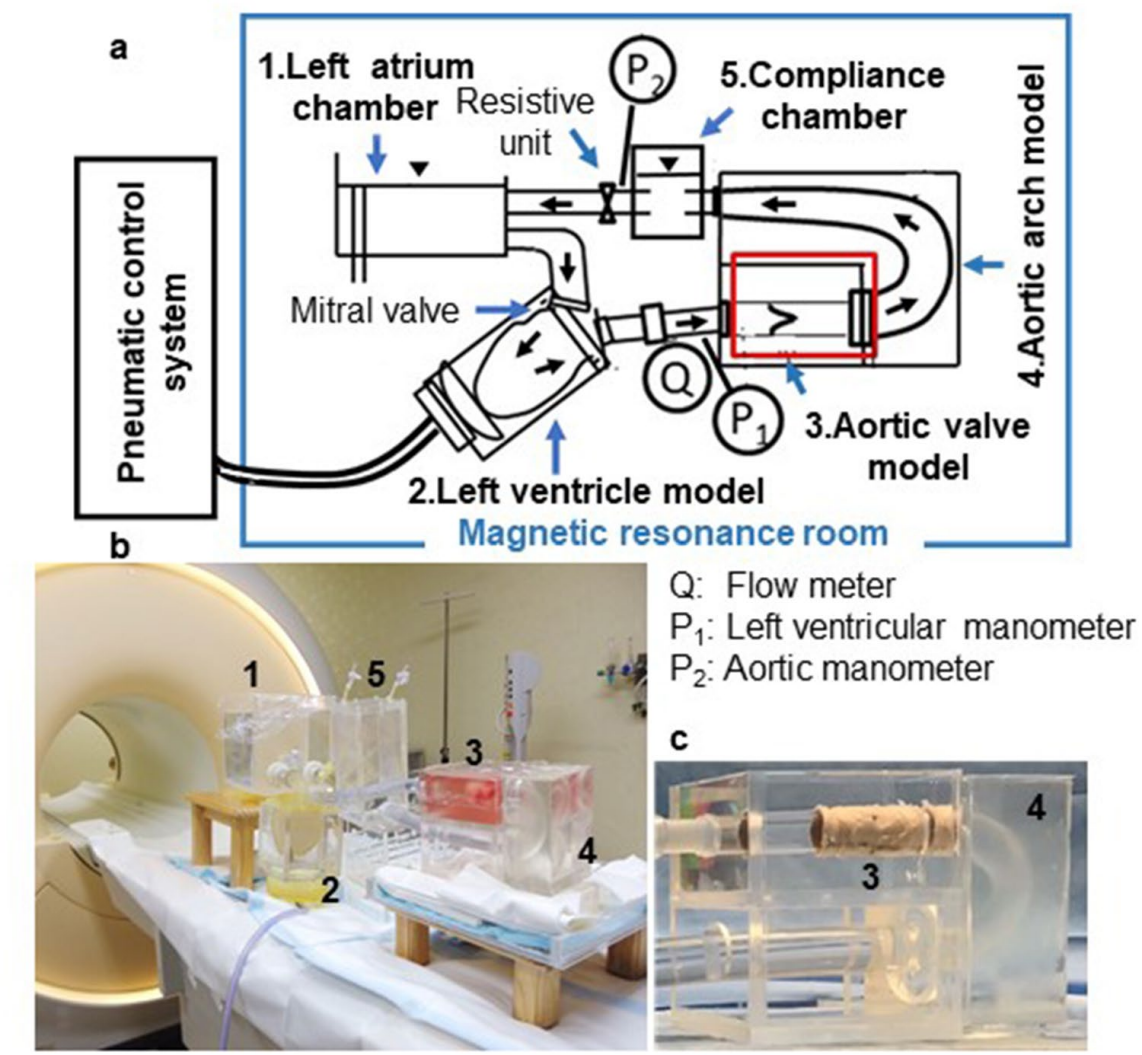
Magnetic resonance imaging (MRI)-compatible pulsatile flow and pressure circulation system. (**a**) MRI-compatible circulation system composed of a left atrium chamber, mitral valve model, left ventricle model, aortic valve model, aortic arch model, compliance chamber, and resistive unit. The pneumatic control system, connected via tubing to a left ventricle model, is placed outside the magnetic resonance room. (**b**) The setup on the magnetic resonance examination table. (**c**) Aortic arch model incorporating an aortic valve model.

**Figure 4:**
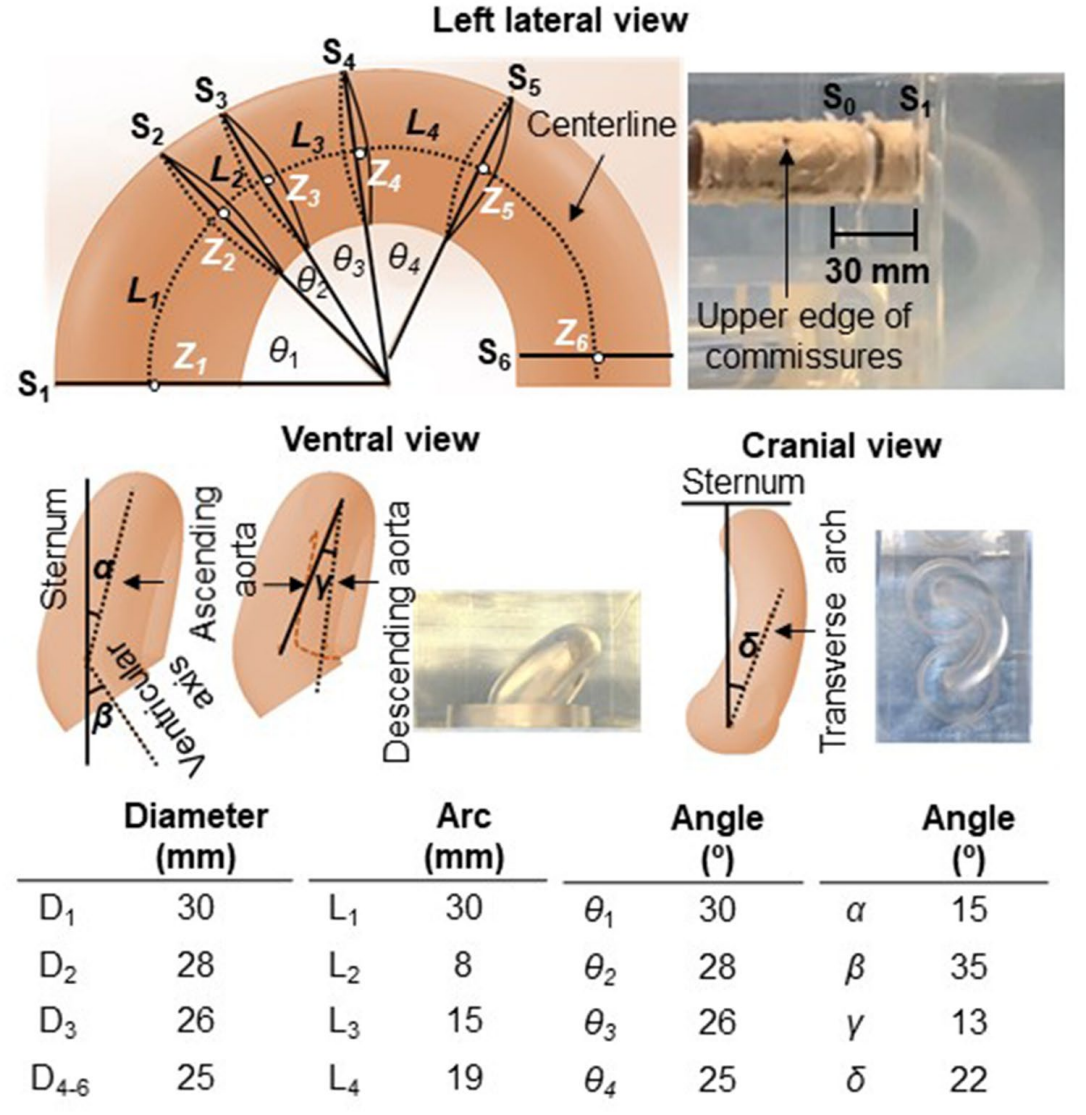
Morphometric and geometric parameters of the aortic arch model. Point Z_1_ is located at the level corresponding to the pulmonary trunk bifurcation. Points Z_2_, Z_3_, and Z_4_ are positioned at the distal edge of the brachiocephalic artery, left common carotid artery, and left subclavian artery, respectively. Points Z_5_ and Z_6_ are located at the level of the upper edge of the body of the fourth and fifth thoracic vertebrae, respectively. Planes S_1–5_ and S_6_ are the cross-sections perpendicular to the aortic centerline at points Z_1–5_ and Z_6_, respectively. Diameters D_1–5_ and D_6_ are aortic diameters of S_1–5_ and S_6_, respectively. Aortic angulations *θ*_1–4_ are measured as the angles between two adjacent planes. Length L_1–4_ are measured for each zone along the centerline. The distance between S_0_ and S_1_ corresponds to the length of the supra-valve part of the aortic valve model (30 mm). The deviation angles of the ascending aorta (α) and ventricular axis (β) to the left of the median plane and that of the ascending aorta with the descending aorta (γ) are measured on a ventral view. The deviation angle of the transverse arch to the perpendicular plane to the sternum (δ) is determined on a cranial view.

The MRI-compatible pulsatile flow circulation system, which duplicated the thoracic aortic circulation, was composed of an elastic left ventricle (LV) model, aortic valve model, aortic arch model, aortic compliance chamber, resistive unit, left atrium pressure chamber, and polymeric valve as an alternative to the mitral valve (Fig. 3a,b). The elastic LV model was operated using a pneumatic control system (VCT-50 (χ), Nippro, Osaka, Japan) to simulate periodically contracting and relaxing myocardium. The aortic arch model with a tunnel-shape (Fig. 3c) was created using silicone (Shin-Etsu Silicone [KE-1603-A: KE-1603-B: KF-96-50CS; 10:10:1], Shin-Etsu Chemical, Tokyo, Japan) to provide a static signal for background phase correction during MRI. The 3D aortic arch model with the geometries based on the literature values of young human aortic arch were devised (Fig. 4)^17,18^. The aortic compliance chamber was installed behind the aortic arch model to simulate arterial pulse pressure. The working fluid, which was physiological saline solution, was recirculated to the left atrium chamber via the peripheral resistive unit. Pneumatic positive pressures in the LV model were adjusted to drive pulsatile flow through the flow circuit at systolic LV pressure of 150 mmHg. A pulse rate of 70 beats per minute (bpm) and systolic fraction of 35% were set in the pneumatic control system.

### Valvular hemodynamics in aortic valve models

Valvular hemodynamics of the aortic valve models, including the mean aortic flow, forward flow, regurgitation (regurgitant volume related to the dynamics of the valve closure), and leakage were measured at a pulse rate of 70 bpm, systolic fraction of 35%, and target aortic pressure of 150/80 (100) mmHg. The measurements were performed using six different models for each aortic valve morphology. The mean values were statistically compared among the six aortic valve morphologies.

### MRI protocol

MRI was performed using a clinical 3.0-T scanner (Phillips Ingenia; Phillips Medical System, Best, The Netherlands) and a 32-element, cardiac phased-array coil for radiofrequency reception. Flow-sensitive 3D gradient sequence was obtained using turbo field echo (TFE) and echo planar imaging (EPI) (TFE factor, 4; EPI factor, 3; repetition time/echo time, 5.7/2.9 ms; flip angle, 15°; velocity encoding, 200 cm/s; fractional field of view, 250 mm × 250 mm; slab thickness, 2.5 mm; matrix, 2.5 × 2.5 × 2.5; spatial resolution, 2.23 × 2.23 × 1.25 mm^3^; frames per cardiac cycle, 14) and an oblique-sagittal slab encompassing the aortic arch model. A simulated electrocardiographic signal with an RR interval of 857 ms, corresponding to a pulse rate of 70 bpm with mean flow of 4.9 ± 0.10 L/min, was used for gating. Scan times ranged from 10 to 15 min (mean, 12 min). Before analyses, the images were corrected for Maxwell phase effects, encoding errors due to gradient field distortions, and eddy current-related phase offsets.

### Qualitative evaluation of eccentric outflow jets at peak systole

Corrected velocity data were imported into 3D visualization software (iTFlow; Cardio Flow Design, Tokyo, Japan) to analyze the fluid flow in the aortic arch model using velocity vector fields. Streamlines, which are instantaneous paths tangential to the velocity vectors at a specific point of time, were visualized at peak systole to assess the characteristics of the aortic valvular outflow jet.

The degree of outflow jet eccentricity, defined as the flow displacement of the high-velocity outflow jet from the center of the vessel, was evaluated in three cross-sections of supra-valve (S_0_), at the level corresponding to pulmonary bifurcation (S_1_), and distal edge of the brachiocephalic artery (S_2_), as described previously^15^. It was graded as follows: no eccentric jet = centrally focused high-velocity systolic flow occupying a majority of the vessel lumen; mild eccentric jet = high-velocity systolic flow occupying between 1/3 and 2/3 of the vessel lumen; and marked eccentric jet = high-velocity systolic flow occupying ≤ 1/3 of the vessel lumen.

### Quantitative analysis of helical flow components at peak systole

The circulation values (m^2^/s) that corresponded to the surface integral values of two-dimensional (2D) vorticity (/s) were calculated at four cross-sections (level corresponding to the pulmonary bifurcation, S_1_; distal edge of the brachiocephalic artery, S_2_; distal edge of the left subclavian artery, S_4_; and fourth thoracic vertebral body upper edge, S_5_) to quantify the magnitude of helical flow components at the four aortic levels, and to investigate the level of the aorta at which helical flow developed, maximized, and faded out. For each cross-section, a 2D vorticity (*ω*) map of time-resolved through-plane vorticity was computed to calculate the circulation value (Γ) according to the following formula:

$$\text{r}=\int_{s} w^{\varvec{.}}\,dS$$

$$ (\omega = rotv = ({{\partial v_{y} \left( t \right)}})/{\partial x} - ({{\partial v_{x} \left( t \right)}})/{\partial y}). $$

The term $v$ $\left( {v_{x} \left( t \right), v_{y} \left( t \right)} \right)$ represents the velocity measured at any time point *t* of the cardiac cycle at a defined anatomical position (*x*, *y*). Positive values represent right-handed rotation, whereas negative values represent left-handed fluid motion as viewed from distal to the aortic flow. The magnitudes of the maximum circulation values at peak systole were obtained from the circulation waveforms computed at the four cross-sections.

### Statistical analyses

Statistical analyses were performed using JMP Pro v15.0 (SAS Institute, Cary, NC, USA). Quantitative variables are expressed as means ± standard deviations. Valvular hemodynamic parameters, including the mean aortic flow, forward flow, regurgitation, and leakage were compared among the six valve morphologies using the Tukey honestly significant difference test. A *P-*value < 0.05 was considered statistically significant.

## Results

### Valvular hemodynamics in aortic valve models

**Figure 5:**
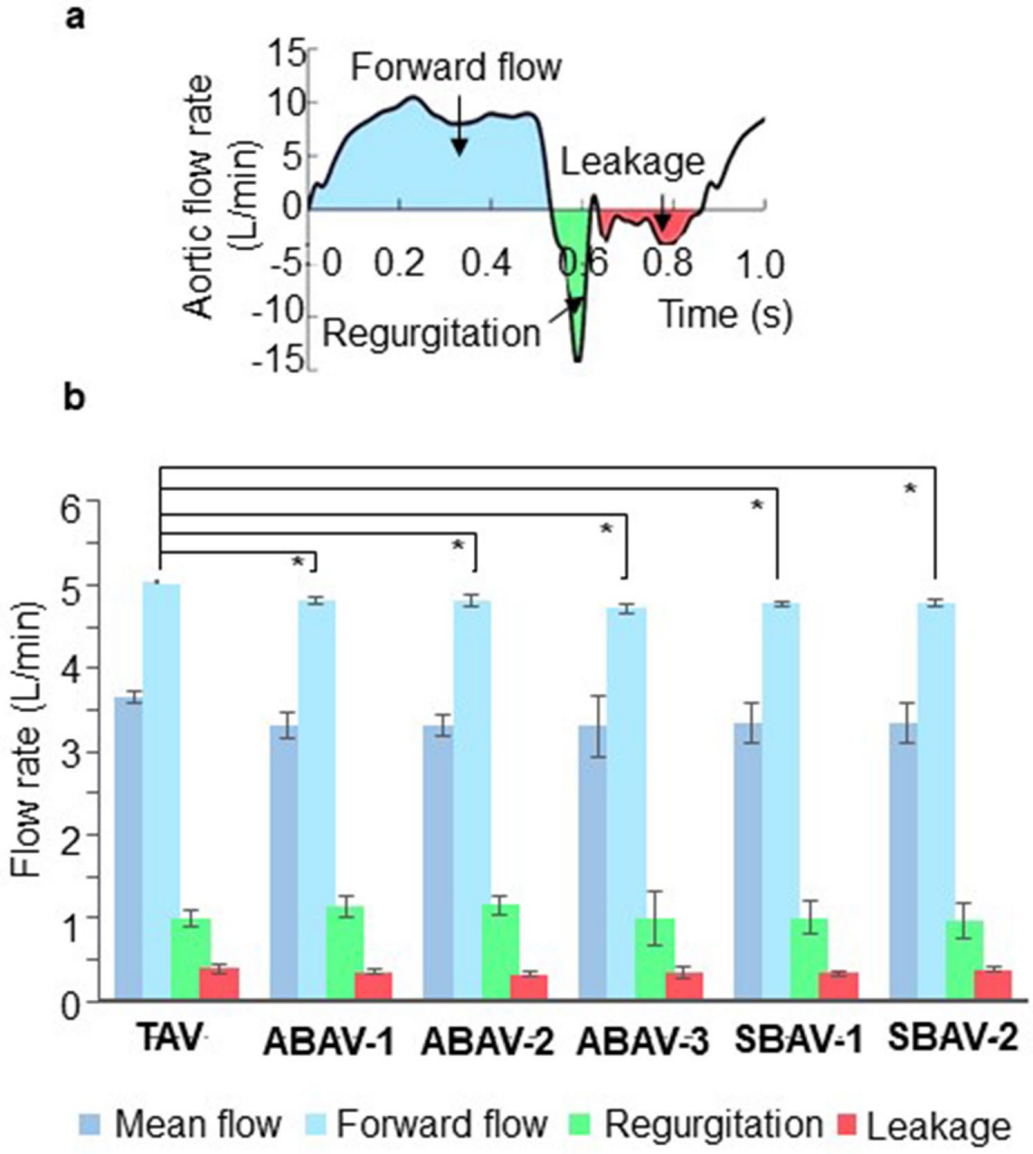
Comparison of the flow among the five bicuspid aortic valves (BAVs) and the tricuspid aortic valve (TAV). (**a**) A representative waveform of the aortic flow rate in TAV and definitions of forward flow, regurgitation, and leakage (blue, green, and red areas, respectively). (**b**) Comparison of the mean aortic flow, forward flow, regurgitation, and leakage among the six valve morphologies (six samples, each). No differences in the mean aortic flow, forward flow, regurgitation, and leakage are observed among the five BAVs. Forward flow is significantly greater in TAV than in BAVs. The mean flow, regurgitation, and leakage are comparable between BAVs and TAV. *Statistical difference (*p* < 0.001, Tukey honestly significant difference test) in comparison of the forward flow between TAV and each BAV. The error bar indicates the mean ± SD.

The aortic flow rate waveform in the TAV is shown as a representative in Fig. 5a. There were no differences in the aortic flow rate curves among the 6 valve morphologies. The peak flow rate was slightly higher in the TAV in comparison with those of the BAVs. Among the five BAVs, no differences were observed in the mean aortic flow, forward flow, regurgitation, and leakage (Fig. 5b). The data of BAVs were comparable to those of the TAV; however, the forward flow in TAV was significantly greater than those in BAVs (see Supplementary Table S1 online).

### Qualitative evaluation of eccentric outflow jets at peak systole

**Figure 6:**
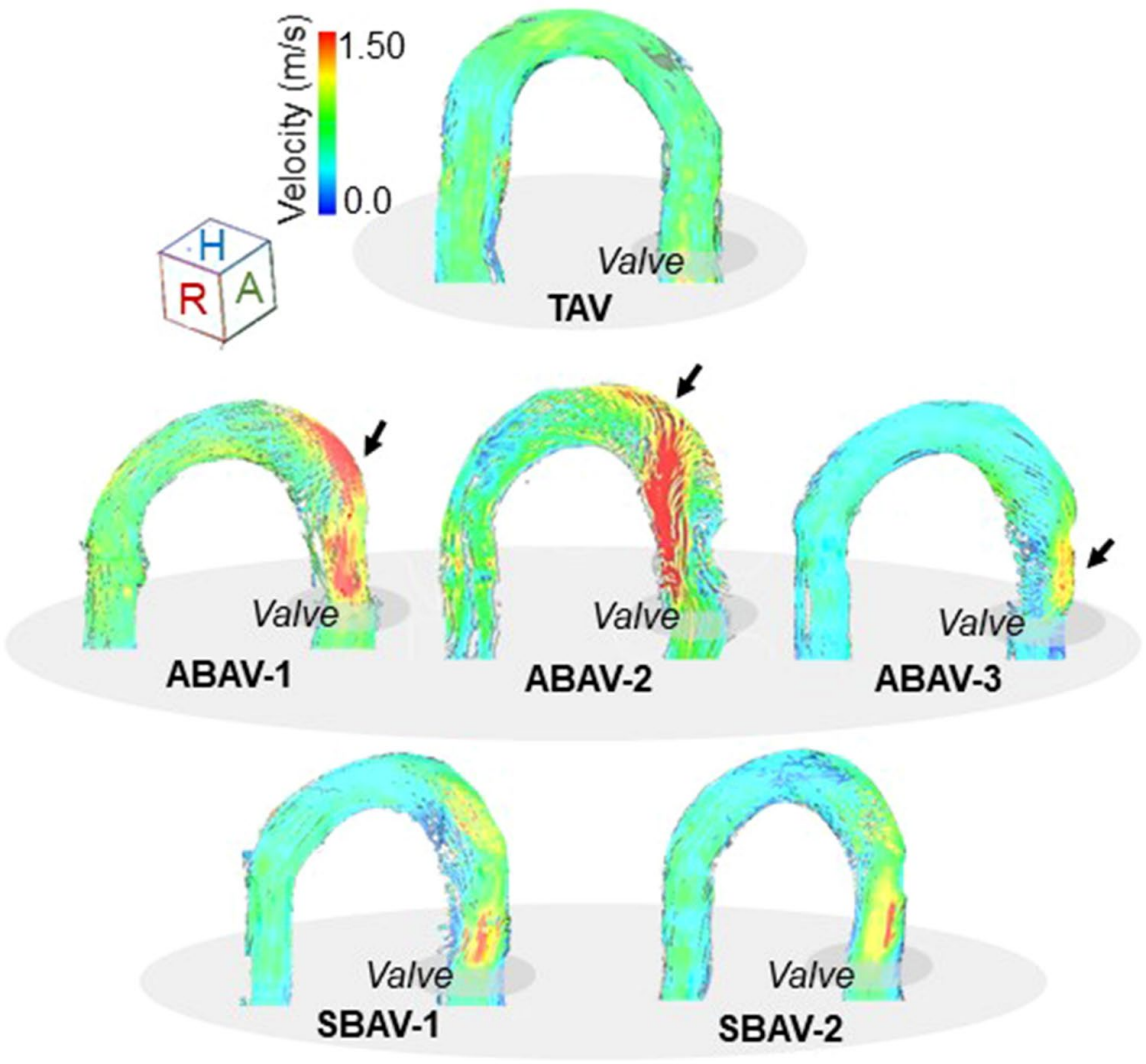
Streamlines of peak systolic aortic flow in the six valve morphologies. The tricuspid aortic valve (TAV) presents a laminar aortic flow. In the asymmetric bicuspid aortic valves (ABAVs), ABAV-1 morphology revealed eccentric aortic valvular outflow jet impinging on the outer curvature of the ascending aorta; ABAV-2 morphology revealed a long-traveling outflow jet impinging on the outer curvature of the proximal transverse arch; and ABAV-3 morphology revealed a short-traveling outflow jet impinging on the supra-valvular aortic wall. In symmetric BAVs, aortic valvular outflow jets do not impinge on the aortic wall. Arrows indicate the aortic wall locations where the aortic valvular outflow jet impinges.

**Figure 7:**
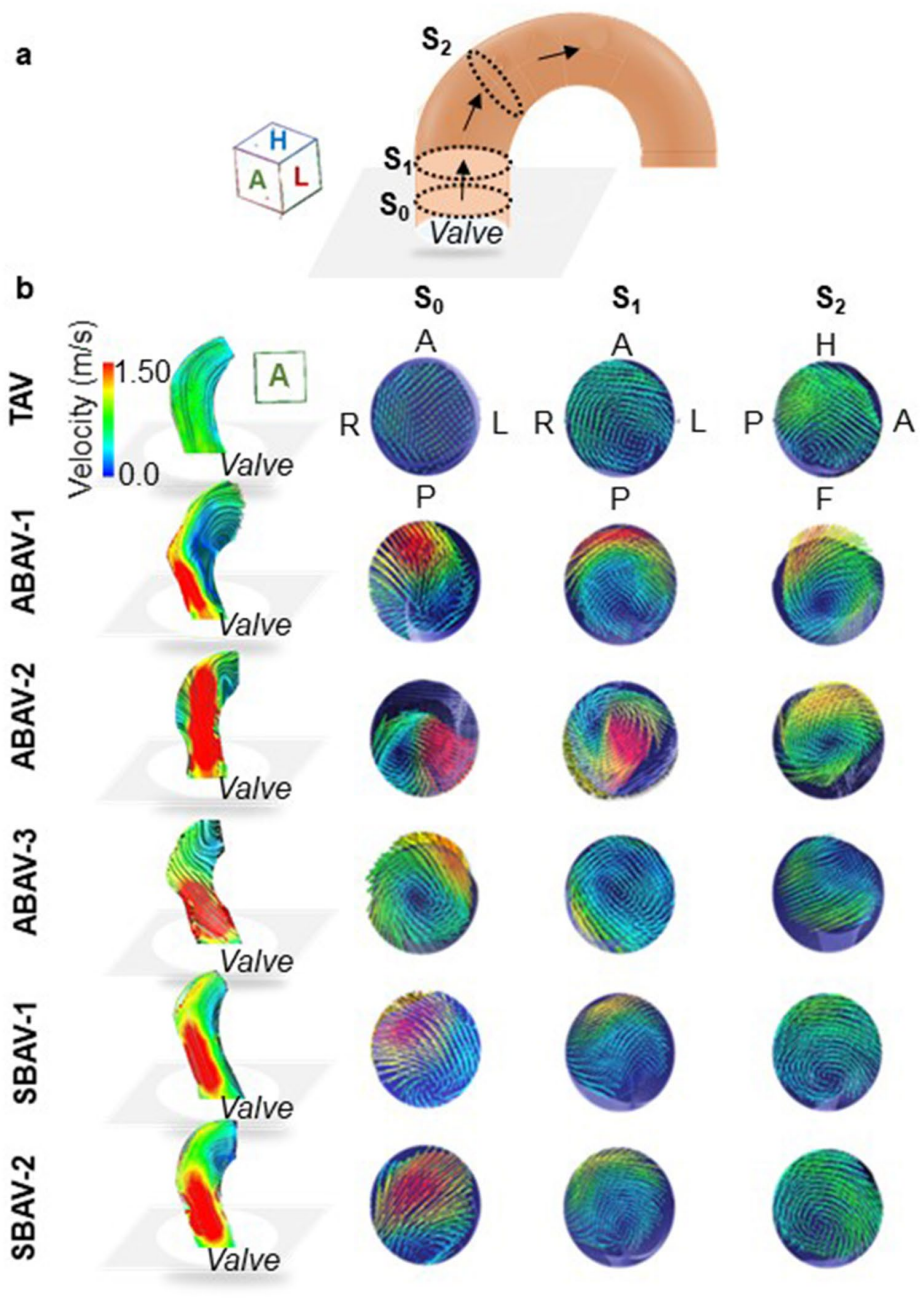
Morphotype-specific aortic valvular outflow jets and the degree of outflow jet eccentricity. (**a**) The position of the three cross-sections: at the level of the supra-valve (S_0_), pulmonary bifurcation (S_1_), and distal edge of brachiocephalic artery (S_2_). (**b**) Left, streamlines of aortic valvular outflow jet at peak systole on a ventral view. Right, cross-sections of the ascending aorta (S_0–2_) demonstrating flow displacement of the aortic valvular outflow jets from the vessel center. Tricuspid aortic valve (TAV) revealed no eccentric flow at peak systole. The three asymmetric bicuspid aortic valves (ABAVs) revealed markedly eccentric peak systolic aortic valvular outflow jets with high-velocity vectors occupying one-third or less of the vessel lumen. The two symmetric BAVs demonstrated mildly eccentric peak systolic aortic valvular outflow jets with high-velocity vectors occupying between 1/3 and 2/3 of the lumen. *A* anterior, *F* foot, *H* head, *L* left, *P* posterior, *R* right.

The BAV models demonstrated morphotype-dependent aortic valvular outflow jets at peak systole (Figs. 6, 7). In the ABAVs, markedly eccentric outflow jets exclusively directed to the aortic wall facing the smaller leaflet were observed. In the ABAV-1, an RA-directed high-velocity outflow jet impinged on the outer curvature of the ascending aorta. In the ABAV-2, an LP-directed high-velocity outflow jet shifting to the proximal transverse arch was observed, and the long-traveling eccentric outflow jet impinged on the outer curvature of the proximal transverse arch. In the ABAV-3, a short-traveling, markedly eccentric outflow jet impinged on the LA quadrant of the ascending aorta at the supra-valve level. In the symmetric BAVs, mildly eccentric outflow jets were present in the ascending aorta. Mildly RA- and LA-diverted high-velocity outflow jets that did not impinge on the aortic wall were observed in the SBAV-1 and SBAV-2, respectively. In the TAV model, laminar flow was observed at peak systole.

### Quantitative analysis of helical flow components at peak systole

**Figure 8:**
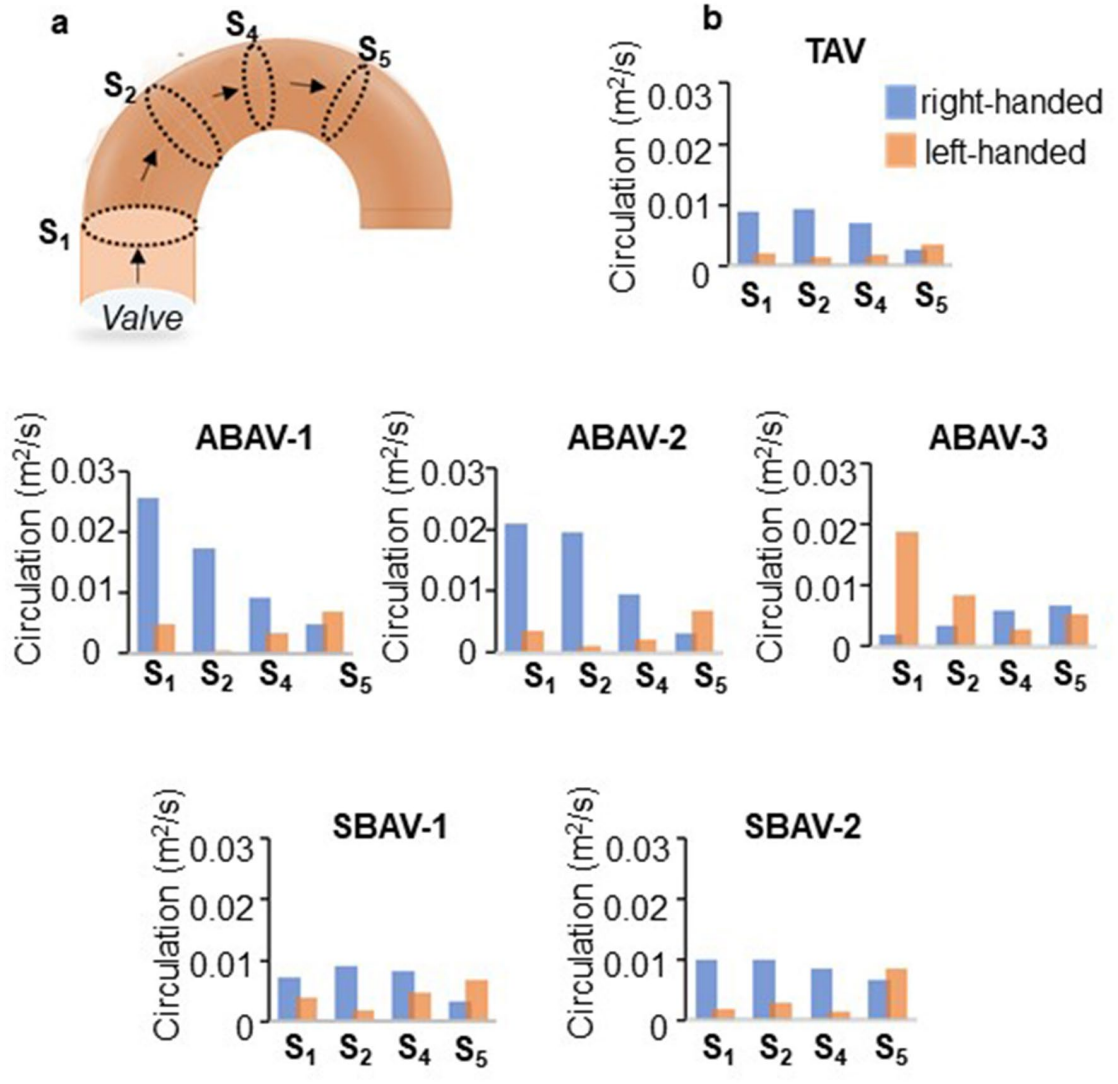
Characteristics of peak systolic circulation values in tricuspid aortic valve (TAV), three asymmetric bicuspid aortic valves (ABAVs), and two symmetric BAVs. (**a**) The position of the four cross-sections: at the level of the pulmonary bifurcation (S_1_), distal edge of brachiocephalic artery (S_2_), distal edge of the left subclavian artery (S_4_), and fourth thoracic vertebra body upper edge (S_5_). (**b**) Magnitudes of maximum circulation values (m^2^/s) at peak systole are measured at S_1_, S_2,_ S_4_, and S_5_ (one sample, each). In the three asymmetric BAVs, the magnitudes of circulation values are markedly high in the ascending aorta, which decrease to approximately half and less than one third in the middle and distal parts of the aortic arch, respectively. In the two symmetric BAVs, the magnitudes of circulation values are comparable to that in TAV.

The maximum values of circulation (m^2^/s) at peak systole measured at the four cross-sections are presented in Fig. 8. In the asymmetric BAVs, marked increases in the magnitudes of circulations were observed in the ascending aorta, which decreased to approximately half and less than one third at the middle and distal parts of the transverse arch, respectively. In the symmetric BAVs, the magnitudes of circulations were comparable to those in TAV throughout the aortic arch model. The predominant rotational directions of the peak systolic helical flow varied according to the BAV morphology; The ABAV-1, ABAV-2, SBAV-1, and SBAV-2 morphologies presented right-handed rotational flow in the ascending aorta, whereas the ABAV-3 morphology presented left-handed rotational flow. Regardless of aortic valve morphology, the predominant rotational directions in the ascending aorta were reversed in the distal arch.

## Discussion

The findings of this study revealed that BAV morphology did not affect the mean aortic flow and leakage, but did significantly alter the aortic wall location where the aortic valvular outflow jet impinged along with the magnitude of circulations at peak systole. The present results indicated that the behavior of the aortic valvular outflow jet depended on the bicuspid symmetry and the position of the smaller leaflet. In asymmetric BAVs, the position of the smaller leaflet affected the impinging portions of the outflow jet on the aortic wall along with the markedly eccentric aortic valvular outflow jets at peak systole. The impinging locations were consistent with the reported regions of increased risk of future aortic events^19^. Furthermore, in asymmetric BAVs, the helical flow components with increased circulations developed in the ascending aorta at peak systole. The predominant rotational directions of the helical flow components varied among the valve morphologies and depended on the direction of the outflow jet as previously reported^20^. The right and posterior directional outflow jets generated right-handed rotations, whereas LA-directed outflow jet in ABAV-3 morphology resulted in left-handed rotation^20^. In symmetric BAVs, the abnormal helical flow did not develop at peak systole but developed mildly eccentric aortic valvular outflow jets that did not impinge on the aortic wall.

These findings provide novel insights into the prediction of risk of aortopathy in BAV. The ABAV-1 morphology, which resulted in a markedly eccentric outflow jet impinging on the aortic outer curvature, might be a risk factor of asymmetric aneurysm bulging toward the outer curvature of the ascending aorta that is uniquely and frequently observed in patients with BAV^21^. The ABAV-2 morphology, which resulted in a long-traveling outflow jet impinging on the outer curvature of the proximal transverse arch, might be a risk factor of aortic aneurysm involving the transverse arch. The ABAV-3 morphology, which resulted in a markedly eccentric outflow jet impinging on the supra-valvular aortic wall, might be a risk factor of aortic aneurysm involving the proximal ascending aorta. In symmetric BAVs, secondary factors, such as valve stenosis, deformation, and aortic dimensions may affect the characteristics of the aortic valvular outflow jet, which are expected to play roles in the development of aneurysms^20,22^. The present results also suggest that aortopathy in BAV may not involve the distal transverse arch, irrespective of the valve morphology.

It has been recognized that BAV morphology, categorized according to the bicuspid fusion pattern and number of raphes^23^, affects the natural history of aortopathy in BAV^24,25^. Recent advances in 4D-flow MRI have suggested that outflow jet eccentricity is a potential index for the aortopathy phenotype in BAV; it was reported that type 3 aortopathy (involving the aortic arch) was more common in the right and non-coronary cusp fusion phenotype (RN-BAV), whereas type 2 aortopathy (dilatation in the ascending aorta) was more frequent in the right and left coronary cusp fusion phenotype (RL-BAV)^14,26,27^. However, due to the anatomical varieties and hemodynamic differences among patients clinically, the relationship between BAV morphology and characteristics of the aortic valvular outflow jet has not been determined. Furthermore, uncommon BAV phenotypes, such as left and non-coronary cusp fusion phenotype (LN-BAV), symmetric BAV, or unclassifiable BAV have been difficult to study clinically.

The present study elucidated the relationships between BAV morphology and aortic valvular outflow jets based on a new classification of BAV morphology using 4D-flow MRI, MRI-compatible pulsatile flow circuit, and different morphological types of BAV models that were developed utilizing 3D-modeling technologies. The characteristics of aortic valvular outflow jet observed in the ABAV-1 and ABAV-2 morphologies were comparable to previously reported 4D-flow MRI findings for RL-BAV and RN-BAV, respectively, probably due to the similarity of the position of smaller leaflet. Furthermore, this study uncovered the hemodynamic features of LN-BAV (mostly included in the ABAV-3 morphology) and symmetric BAV, which have hardly been observed clinically. The classification scheme for BAV morphology proposed in the present study was based on two characteristics that directly affected the location of the outflow jet impinging on the aortic wall—the symmetry and position of the leaflets. The spatial position of the leaflets was defined based on the aortic quadrant faced by the smaller leaflet independent of the position of the raphe, commissures, and coronary artery orifices. Our classification of BAV morphology may be useful for predicting the risk of aortopathy in BAV.

To the best of our knowledge, this is the first report to demonstrate the effects of BAV morphology on aortic hemodynamics using an MRI-compatible pulsatile flow circulation system. Several studies have experimentally investigated aortic hemodynamics using an MRI-compatible pulsatile flow circuit^28,29^. However, none of them included MRI-compatible pulsatile flow circulation system that incorporated different morphological types of tissue-based aortic valve models.

The methodology of modeling aortic valves using morphotype-specific valve-devising apparatuses enabled the creation of several BAV models with reproducibility. The analysis of hemodynamic features in uncommon BAVs was performed using the valve-devising technique. Furthermore, the morphologically relevant aortic arch model, designed to minimize artifacts during MRI, enabled the visualization and quantification of 4D-flow MRI data with sufficient quality. The MRI-compatible pulsatile flow circulation system developed in the present study may be useful for studying valve-related aortic hemodynamics, especially in uncommon valve phenotypes that are rarely encountered clinically.

The first limitation of the present study is the relatively large regurgitation, which was possibly due to the absence of the sinus of Valsalva and the lack of the closing force of the sinus vortices. However, a tubular aorta with predefined dimensions, not an aortic root, was intentionally used to create aortic valve models to maintain the uniformity and reproducibility of valve modeling. Regurgitation was maintained under 30% in forward flow, which was considered clinically acceptable in each morphology. Second, the 3D helical flow parameters were analyzed at the prescribed cross-sections over the aortic arch model. The major helical flow components, such as rotation around an axis parallel to the main flow, could be identified and quantified in this study. Third, the physiological saline solution used as the working fluid of the pulsatile flow circulation system did not reflect the viscosity of blood in human body. The use of saline solution might result in a relatively higher Reynolds number. However, the aortic flow belongs to the highly turbulent flow regime, where the effects of the viscosity is negligible^30^. We believe that the relative differences between the present results and in-vivo findings are expected to be small. Finally, because the present study only aimed to demonstrate the effects of BAV morphology on aortic hemodynamics, the effects of other factors, such as valve stenosis, deformation, and aortic arch dimensions were simplified. The effects of these factors on hemodynamics will be studied in the future.

In conclusion, we elucidated the relationships between BAV morphology and characteristics of aortic valvular outflow jet using an MRI-compatible pulsatile flow circulation system for the first time. This study revealed that the bicuspid symmetry and the position of smaller leaflet in BAV were determinant factors of the characteristics of the aortic valvular outflow jet. Asymmetric BAV morphology with a smaller leaflet facing the right anterior quadrant of the ascending aorta may be a risk factor of asymmetric aneurysm bulging toward the outer curvature of the ascending aorta. Asymmetric BAV morphology with the smaller leaflet facing the left posterior quadrant of the ascending aorta may be a risk factor of aortic aneurysm involving the transverse arch. Asymmetric BAV morphology with the smaller leaflet facing the left anterior quadrant of the aorta may be a risk factor of aortic aneurysm involving the proximal ascending aorta. Symmetric BAV morphologies may contribute to the progression of aortopathy along with other factors that affect the aortic valvular outflow jet. Our classification of BAV morphology may be useful in predicting the risk of aortopathy in BAV.

## Supplementary Information

Supplementary Information.
