# Supplementary material for: Bicuspid aortic valve morphology and aortic valvular outflow jets: an experimental analysis using an MRI-compatible pulsatile flow circulation system
Source: Sci Rep. 2021 Jan 22;11:2066. doi: 10.1038/s41598-021-81845-w (PMC7822932; doi:10.1038/s41598-021-81845-w)
Supplement: Supplementary file 1 — Supplementary Information. [file 41598_2021_81845_MOESM1_ESM.pdf]

Bicuspid Aortic Valve Morphology and Aortic Valvular Outflow Jets: An Experimental Analysis Using an MRI-Compatible Pulsatile Flow Circulation System

Kaoru Hattori, Natsuki Nakama, Jumpei Takada, Gohki Nishimura, Ryo Moriwaki, Eita Kawasaki, Michinobu Nagao, Yasuhiro Goto, Hiroshi Niinami, and Kiyotaka Iwasaki

Mean flow (L/min) (n = 6, each)		*P* value *				
		ABAV-1	ABAV-2	ABAV-3	SBAV-1	SBAV-2
TAV	3.66 ± 0.07	0.15	0.18	0.16	0.24	0.27
ABAV-1	3.31 ± 0.15		1.00	1.00	1.00	1.00
ABAV-2	3.32 ± 0.13			1.00	1.00	1.00
ABAV-3	3.31 ± 0.37				1.00	1.00
SBAV-1	3.34 ± 0.23					1.00
SBAV-2	3.35 ± 0.24					

Forward flow (L/min) (n =6, each)		*P* value *				
		ABAV-1	ABAV-2	ABAV-3	SBAV-1	SBAV-2
TAV	5.04 ± 0.02	<0.0001	<0.0001	<0.0001	<0.0001	<0.0001
ABAV-1	4.81 ± 0.04		1.00	0.087	0.82	0.97
ABAV-2	4.80 ± 0.08			0.11	0.87	0.98
ABAV-3	4.72 ± 0.06				0.63	0.36
SBAV-1	4.77 ± 0.04					1.00
SBAV-2	4.78 ± 0.05					

Regurgitation (L/min) (n = 6, each)		*P* value *				
		ABAV-1	ABAV-2	ABAV-3	SBAV-1	SBAV-2
TAV	0.99 ± 0.01	0.85	0.75	1.00	1.00	1.00
ABAV-1	1.14 ± 0.12		1.00	0.82	0.89	0.77
ABAV-2	1.16 ± 0.11			0.72	0.80	0.66
ABAV-3	0.99 ± 0.32				1.00	1.00
SBAV-1	1.00 ± 0.20					1.00
SBAV-2	0.98 ± 0.21					

Leakage (L/min) (n = 6, each)		*P* value *				
		ABAV-1	ABAV-2	ABAV-3	SBAV-1	SBAV-2
TAV	0.39 ± 0.06	0.97	0.38	0.65	0.54	1.00
ABAV-1	0.36 ± 0.04		0.83	0.98	0.94	1.00
ABAV-2	0.33 ± 0.03			1.00	1.00	0.61
ABAV-3	0.34 ± 0.08				1.00	0.86
SBAV-1	0.34 ± 0.03					0.78
SBAV-2	0.38 ± 0.03					

Supplementary Table S1. Comparison of the flow among the five bicuspid aortic valves (BAVs) and the tricuspid aortic valve (TAV). Data of the flow indicates the mean ± standard deviation. * By Tukey honestly significant difference test.
